# Use of Standardized Patient Simulations to Assess Impact of Motivational Interviewing Training on Social–Emotional Development

**DOI:** 10.3390/pharmacy6030065

**Published:** 2018-07-11

**Authors:** Suzanne Galal, Deepti Vyas, John Mayberry, Edward L. Rogan, Shivani Patel, Sara Ruda

**Affiliations:** 1 Pharmacy Practice Department, Thomas J. Long School of Pharmacy and Health Sciences, University of the Pacific, Stockton, CA 95211, USA; sgalal@pacific.edu (S.G.); erogan@pacific.edu (E.L.R.); s_patel54@u.pacific.edu (S.P.); s_ruda@u.pacific.edu (S.R.); 2 Department of Mathematics, College of Pacific, University of the Pacific, Stockton, CA 95211, USA; jmayberry@pacific.edu

**Keywords:** simulation, standardized patients, smoking cessation, active learning, mock consultation

## Abstract

The objective of this study was to assess the impact of motivational interviewing (MI) training on students’ social–emotional development. Two simulations using standardized patients (SP) were conducted within a smoking cessation module. Students first completed a 4 h self-study module focused on smoking cessation tools and general counseling techniques. Faculty then administered a 15-item rubric focused on students’ self-assessment of their verbal/non-verbal communication, social–emotional competence and MI skills. Students then participated in a smoking cessation counseling session with an SP. SPs used the same rubric to assess student performance. Teaching assistants (TAs) observed and assessed the students using the same rubric and an additional 22 items related to clinical skills. TAs and SPs then provided feedback on areas of improvement. The following week, students first completed a 3 h self-study module on MI then participated in a different smoking cessation scenario. After completion, the 15-item self-assessment rubric was administered. There was a significant improvement in TA assessed student performance with an average score improvement of 8% (pre-intervention score = 67%; post-intervention mean = 75%). Students had dramatic gains in their self-assessment with their scores rising by an average of 22%. Using MI techniques can improve students’ self-assessed and perceived social–emotional competency.

## 1. Introduction

The 2016 Accreditation Council for Pharmacy Education (ACPE) standards stress the importance of developing professional skills which allow for effective interpersonal dialogue and understanding, to not only provide patient-centered care but also serve as a patient advocate [1]. In addition, the standards propose that pharmacy school programs implement techniques to develop students’ self-awareness so that they may be aware of their own biases, emotions, and motivations and in turn, use that self-awareness to provide optimal patient-centered care [1]. This requires pharmacy schools to identify targeted strategies for developing these skills longitudinally across the curricula. While development of interpersonal communication and overall social–emotional competence is an expectation of all pharmacy graduates, it is still unclear how well these skills are developed and assessed in the pharmacy curricula.

A study conducted by Kimberlin examined behavioral assessment forms used to evaluate pharmacy student counseling skills [2]. They surveyed 50 pharmacy schools and found that only 56% assessed whether a student was able to practice empathy and respond to a patient’s feelings, which is a critical factor in providing patient-centered care [2]. The overall construct of social–emotional competence includes self-awareness of one’s emotional state, empathy for and consideration of the patient, ability to establish a connection, and ability to influence the patient to elicit a change in behavior. Hojat and colleagues demonstrated that healthcare professionals who scored higher on the Jefferson Scale of Empathy had enhanced clinical outcomes for their patients [3]. In their study, Hojat showed that physicians with higher empathy scores had greater control of hemoglobin A1c levels versus physicians with lower empathy scores (56% versus 40% respectively) [3].

It is clear that targeted strategies must be employed to further students’ skills in communicating with patients in a manner which promotes trust and interpersonal relationships. One such strategy is motivational interviewing (MI) which is a counseling technique that can be used to elicit a change in behavior. The 2013 Center for the Advancement of Pharmacy Education, CAPE, outcomes specifically call for pharmacy graduates to demonstrate empathy and the ability to persuade a patient to take control of their health. [4]. CAPE goes one step further and highlights MI as one of the techniques which can be used to build strong interpersonal relationships between the provider and patient [4]. MI is a technique that relies on specific assumptions. One of the assumptions of MI is that “An empathic, supportive, yet directive, counseling style provides conditions under which change can occur” [5]. This style of counseling focuses on a non-judgmental approach to encouraging patients to recognize their ambivalence toward change and utilizing the patient’s intrinsic values to elicit change [6]. 

Many health-related programs have started incorporating MI training to enhance students’ patient counseling skills. In a one-month study with final-year undergraduate nutritionists, researchers sought to assess the efficacy of MI training on students’ empathy and counseling abilities [7]. The study showed that nutritionists who were trained to practice MI had higher “global” scores for empathy and asked more open-ended questions [7]. According to Miller and Rollnick, the founders of MI, open-ended questions allow the provider to further investigate the patient’s mindset and focus their counseling towards those targets [6]. First and third-year medical students at the University of Virginia School of Medicine were taught MI through a series of lectures and small group teaching [8]. Medical students were evaluated through a videotaped interview and an SP who portrayed a smoker. The interview was rated using the Motivational Interviewing Treatment Integrity scoring tool (MITI), which included empathy, spirit, adherence, non-adherence, the types of questions, and the number of reflections [8]. Results showed that medical students reached a proficient level on the rate of reflections after the training, but not on the other measures on the MITI [8]. However, in another measure, the majority of medical students felt more comfortable communicating with patients to initiate behavior change after this training [8]. 

A recent review article emphasized the importance of teaching emotional skills to medical students and highlighted the importance of building effective provider–patient communication [9]. The paper further noted that emotional skills could be developed by targeted educational interventions [9]. Goggin and colleagues found similar results in an elective course for PharmD students [10]. The course was focused on teaching students MI skills and applying these skills to simulated patient interviews [10]. Based on the skills rating forms, they found that students had higher scores at the end of the course and increased self-confidence in their MI skills [10]. 

The impact of MI-based counseling on actual patient outcomes also appears to be mounting. In a study on medication adherence, Abugosh and colleagues showed that Type 2 diabetic patients who received MI-based telephone intervention by pharmacy students had better medication adherence compared to the control arm [11]. Patients who received two or more calls in a six-month period, not only had better medication adherence but also had a smaller likelihood of discontinuing their therapy [11]. Researchers found that dental students who used MI techniques had patients with increased self-efficacy of interdental cleanings compared to patients who received care from dental students who did not use MI techniques [12]. However, despite growing evidence, the exact strategy for training students on MI is still unclear. The use of simulations with SPs may be a useful modality for training students especially if formative feedback is built into the simulations. In a study on MI, researchers compared three different strategies (mock patients, written dialogue, and peer role play) to determine the best to teach MI [13]. The researchers concluded that students that learned from mock patient or SPs had the highest performance on a summative evaluation measuring their effectiveness in using MI techniques [13]. It is also unclear whether MI can increase a student’s social–emotional development (SED). The objective of this study is to assess the impact of MI training on SED with the use of SP simulations within a smoking cessation module.

## 2. Materials and Methods 

First-year students enrolled in a required skills course titled Practicum 1 were enrolled in the study. Students completed a 4 h self-study module created by faculty using materials from the Rx for Change curriculum available at rxforchange.ucsf.edu. This module covers smoking cessation background, the basics of addiction pathophysiology, pharmacological aids, and the 5 As counseling technique (Ask, Advise, Assess, Assist, Arrange). The following week students completed a self-assessment using a 15-item grading rubric to measure their confidence in their communication skills, both verbal and non-verbal, social–emotional competence (SEC) and MI skills. Areas specific to SEC included “self-awareness, consideration, connection, and influence” while areas specific to MI included “partnership, acceptance, compassion, and evocation.” Each of the sections used a 0–3 point scale; 0 = incomplete/inappropriate, 1 = development/needs work, 2 = threshold/meets expectations, 3 = competent/exceeds expectations. The rubric was developed by faculty to serve as an objective assessment tool as well as guide students and assessors in showing what is expected with examples of demonstrative behavior for each of the factors within each scoring category. A total of 15 SPs, who were paid actors, and 15 teaching assistants (TAs) were recruited for this activity. All 15 TAs and SPs participated in both simulations.

Fifteen stations were set up with one TA (observer) and one SP per station. Students then completed a 15 min mock counseling smoking cessation session with SPs playing the role of a smoker. Before the simulation, SPs were trained using character bios created by faculty. The character bios provided in-depth insight into the patient’s personality, intrinsic reasons for smoking and reasons for wanting to quit smoking. SPs received 2 h of training focused on how to use the character bios, how to remain within character, how to use the 15-item grading rubric, and how to provide constructive feedback. TA training included 1 h of instruction on using the grading rubrics as well as how to provide constructive feedback to the students. The grading rubrics were the same across both the SPs and TAs. However, the TA rubric contained additional items on clinical knowledge to assess the accuracy of the information provided by the students (37 items total). Questions on the TA rubric were split along seven dimensions of performance: Introduction (Intro), the 5 As (A5), Medication Consulting (Counsel), Closing (Closing), Verbal/Nonverbal Communication (Comm), SEC, and MI. The SPs were instructed to note the student’s interpersonal communication skills and also provide feedback on their perceptions from a patient’s point of view. For example, SPs were trained to start each feedback point with “As the patient I felt…”

After the first simulation, the students completed a 3 h self-study on MI. The training was developed by faculty to keep students engaged throughout. The material was broken up into eight small modules taking 5–15 min to complete which included videos, images, readings, charts and recourses to look through. After each module, students were asked to apply their knowledge to various reflection questions and written practice scenarios to review the material. While their responses did not get graded, there were examples provided for them to compare their answers. The training was developed to enhance student use of MI techniques within any counseling context not just smoking cessation. 

The following week, students were re-assessed in a simulation similar to the previous week’s assessment. Overall, two separate cases were developed and were different each week with different patient history, personality, demographics, and personal motivations. Again, students were re-assessed by both SPs and TAs. At the conclusion of the second simulated counseling session, students completed the post-assessment using the 15-item grading rubric to determine any changes in their self-perceived communication skills and SEC. Students were instructed not to share any information regarding the case with their classmates. At the end of week 2, students also completed a 16-question survey measuring their perceptions of the simulated experience and the SP feedback on their performance. Students scored various aspects of the experience on a 1–5 Likert scale. In addition, TAs were instructed to rate the SPs performance as a patient to provide some insight into the training as well as an individual performance by each SP.

**Figure 1:**
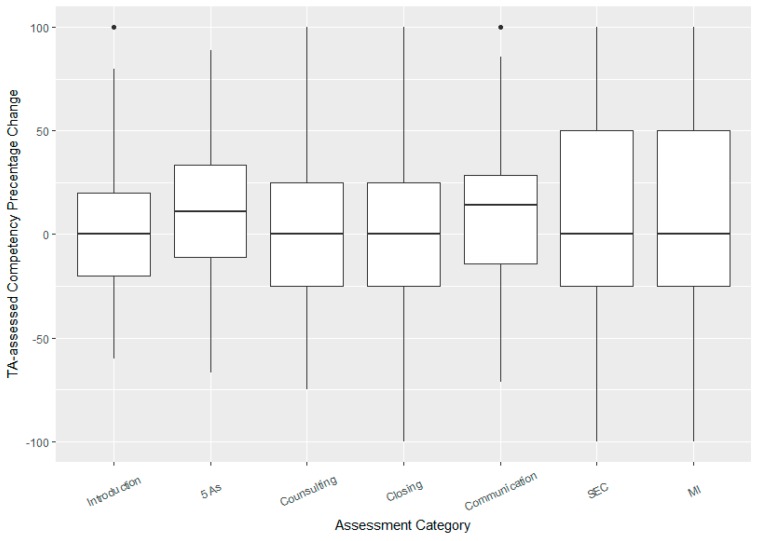
Boxplot showing median improvements in competency scores as assessed by Teaching Assistants (TAs). Box and whiskers show the interquartile range (IQR) and range (excluding outliers), respectively. Points show outliers as identified by the usual “1.5 × IQR” rule.

A paired *t*-test was used to test for a significant mean improvement in competency scores from week 1 to week 2. A “competency score” was defined as the percent of items from the assessment on which the student was scored competent (score = 3). Significant improvements were identified using paired *t*-tests with a Holm-Bonferroni adjustment for multiple comparisons. Due to the presence of some positively skewed distributions (e.g., see SEC and MI in Figure 1), Wilcoxon Tests for changes in median competency scores were also performed, and agreement insignificance was found in all subcategories. Pearson’s product–moment Correlation was used to measure the correlation between improvement in MI and improvement in SEC. All results below are reported with the mean changes along with 95% confidence interval (CI), either in the form (Lower–Upper%) for competency scores or (Lower, Upper) for other quantities, as well as adjusted *p*-values when appropriate. Results with adjusted *p*-values of less than 0.05 were considered statistically significant. Simulated experience scores from week 1 to week 2 were compared with a paired *t*-test and correlated with changes to student competency scores using Pearson’s product–moment correlation. Student responses to these questions were averaged to give a simulated experience score. A similar analysis was applied to the TA evaluations of the SPs (average of 3 Likert scale questions) and students’ overall satisfaction with the experience (average of 6 Likert scale questions). All data analysis was performed using R, v 3.4.3 with a sample size of *n* = 205 students for all analyses except where otherwise indicated in the text. This study was approved by the University of the Pacific Institutional Review Board (IRB). 

## 3. Results

### 3.1. TA Assessment of Students

A total of 205 students participated in this study. Overall, there was a significant improvement in TA assessed student competency scores between the first and second-week assessments with an average student showing a competency score improvement of around 8% (week 1 mean competency = 67%; week 2 mean competency = 75%, t_204_ = −4.491, *p*-value < 0.001). Overall, 55.1% of all students showed some improvement with a 75th percentile improvement of 27.3%. 

On the TA rubric, four of the seven categories showed significant mean improvements in “competency scores”: Introduction (mean improvement = 4% (2–7% CI), adjusted *p*-value = 0.018), 5 As (mean improvement = 11% (7–16% CI), *p*-value < 0.001), Communication (mean improvement = 8% (4–12% CI), *p*-value = 0.002), and SEC included 4 factors (awareness, consideration, connection, influence) (mean improvement of total score = 9% (2–16% CI), *p*-value = 0.032). The boxplots show that the median improvement was also largest for the 5 As and communication (Figure 1). 

**Figure 2:**
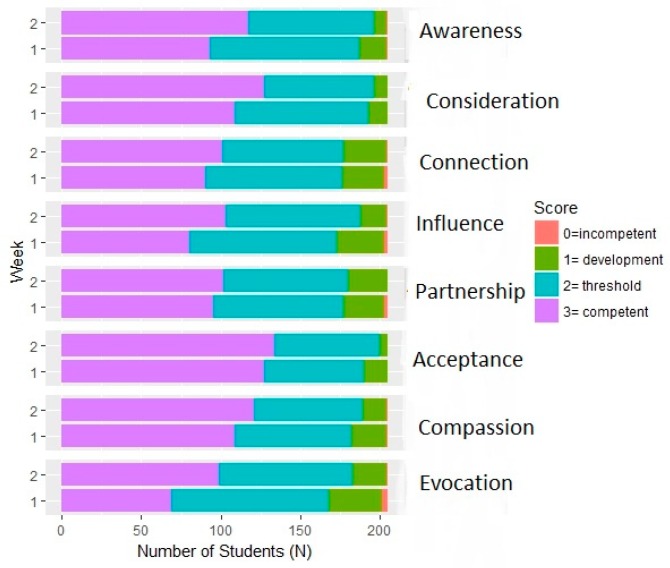
TA assessment of student performance on social–emotional competence and motivational interviewing items (pre and post-intervention).

There was a strong positive correlation between TA assessed student improvements in SEC and improvements in MI with a correlation coefficient of *r* = 0.73 (0.65, 0.78). Using a stacked barplot to further visualize improvements in individual components of these areas, there were increases in the percentage of competent students across all eight areas related to SEC and MI, but the largest gains appeared to be in Awareness, Consideration, Influence, and Evocation (Figure 2).

### 3.2. SP Assessment of Students

**Figure 3:**
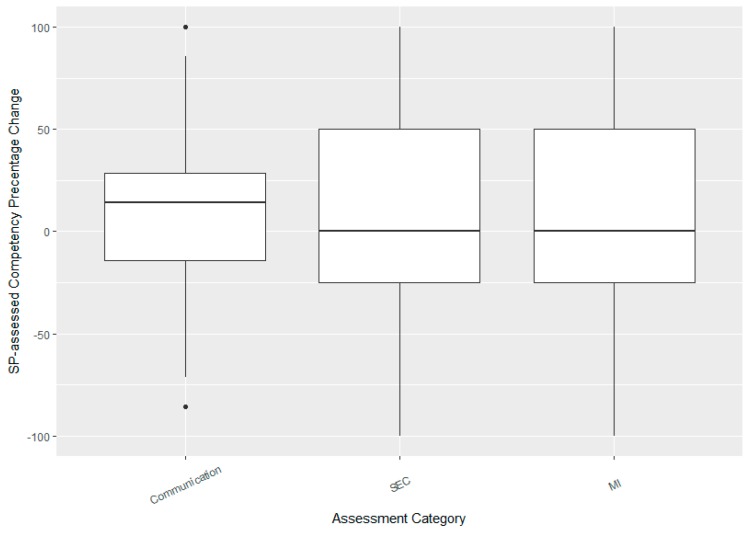
Boxplot showing median improvements in competency scores as assessed by standardized patients (SPs). Box and whiskers show the IQR and range (excluding outliers), respectively. Points show outliers as identified by the usual “1.5 × IQR” rule.

There was a significant improvement in SP assessed student competency scores as well with an average improvement of about 10% (week 1 mean score = 45%; week 2 = 55%; t_204_ = −3.18, *p*-value = 0.002). The plots compare improvements in the three categories (Figure 3). Interestingly, the mean improvements in all three categories were significant (mean increase in communication skills = 9% (4–13%), adjusted *p*-value = 0.001; mean increase in SEC = 8% (1–15%), *p*-value = 0.031; mean increase in MI = 9% (2–16%), *p*-value = 0.031). The median improvement in communication was much higher than in the other two categories due to the skewness of the distributions (Figure 3). As with TA scores, there was a strong correlation between improvements in the two areas of SEC and MI (*r* = 0.79, (0.73, 0.83)).

### 3.3. Comparison of Student, SP and TA Assessments

There was relatively low agreement in ratings between SPs and TAs (Intraclass Correlation Coefficient, ICC2 = 0.52; 0.32, 0.66). Additionally, initial student self-assessments also showed no correlation with either SP or TA assessments (ICC2 with TA = 0.06; ICC2 with SP = 0.14) while their post assessments were only weakly correlated (ICC2 = 0.36 with TA; ICC2 = 0.34 with SP). On average, the SPs tended to rate students significantly lower than TAs with mean differences in competency scores of 14% (10–18%) for week 1 and 13% (9–17%) for week 2. Students were, in general, harder on themselves than either TAs or SPs especially in their initial assessments, which were, on average, 25.5% lower than the TAs (20–31%). Their final assessments, however, were only 10% lower (6–15%) on average than TAs but continued to differ significantly from the assessments given by the SPs during week 2. 

### 3.4. Student Self-Assessment

**Figure 4:**
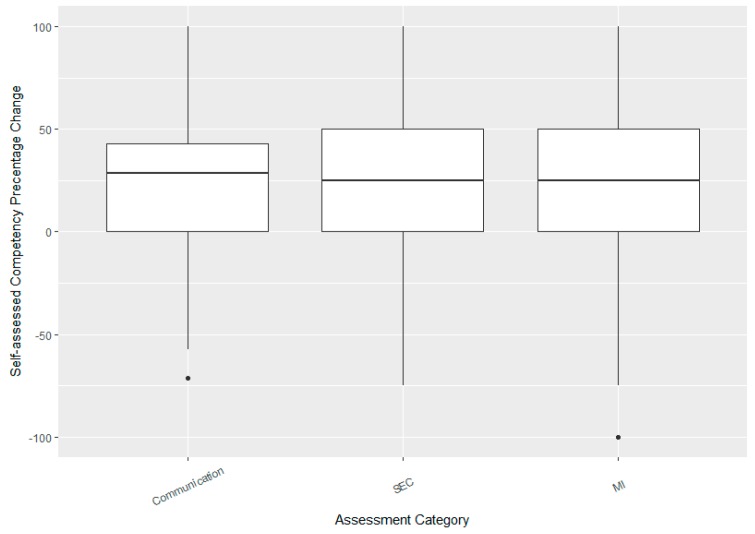
Boxplot showing median improvements in student self-assessed competency scores. Box and whiskers show IQR and range (excluding outliers), respectively. Points show outliers as identified by the usual “1.5 × IQR” rule.

Students showed a dramatic gain in their self-confidence with their post-week two self-reported competency scores rising by an average of 22% (18–27%) from their pre-survey assessments. The plots below compare improvements in Comm, SEC, and MI which were all highly significant (mean change in Comm = 23% (19–27%), SEC = 19% (13–27%), MI = 22% (17–29%), all *p*-values < 0.001). Median improvements were similar across the three categories with roughly symmetric change score distributions (Figure 4). Students also showed a strong correlation between self-reported improvement in SEC and MI (*r* = 0.65 (0.55, 0.72)). 

### 3.5. Student and TA Evaluation of Simulated Experience

Only one hundred and eighty-six (response rate 90.7%) students completed all questions on the perception survey and were included in the analysis. Overall, student opinions of the simulated experience were significantly higher in week 2 than week 1 (mean week 1 = 4.42/5, mean week 2 = 4.54/5, CI for diff = (0.04, 0.22), t_178_ = 2.90, *p*-value =0.004). 

There did not appear to be any significant correlation between student improvement in overall TA assessed competency scores and their opinion of the experience (*r* = 0.11 (−0.04, 0.25), *p*-value = 0.163) (Table 1). Similarly, TA ratings of SPs were significantly higher in week 2 (mean week 1 = 4.79/5, mean week 2 = 4.97/5, CI for diff = (0.11, 0.25), t_204_ = 5.15, *p*-value < 0.001), but were not correlated with TA assessed student improvements (*r* = −0.02 (−0.16, 0.11), *p*-value = 0.754). Students’ overall ratings of the experience were only weakly, positively correlated with TA assessed improvements (*r* = 0.15 (0.01, 0.29), *p*-value = 0.041). Table 1 displays the summary statistics with a breakdown of the post-survey questions and response of students to the overall simulated experience.

## 4. Discussion

In this study, students were assessed by SPs and TAs on several core competency items. Overall, there was a significant improvement in student competency scores between the first and second-week assessments. The most substantial gains were seen in the categories of the 5 As, verbal/nonverbal communication, and SED. These results are consistent with the results from data in other published studies proving to be significant enough for practical purposes. For example, third-year Yale medical students who were taught MI techniques with the use of SPs also demonstrated positive results after a brief MI training, and they maintained these skills after a four week follow up [14].

Our results showed that SP evaluations of the student consultations tended to be more critical than the TA evaluations, suggesting that the SPs perceptions of emotions, empathy and relationship building may differ from the TAs who may be more likely to emphasize clinical content versus interpersonal skills. This stresses the importance of multiple assessment/observations to allow for feedback from various perspectives. In turn, the students’ self-evaluations were the lowest compared to SPs and TA evaluations. Although students were able to view the rubric in advance, they were not trained on the use of the grading rubric, unlike the TAs and SPs who received formal training on the use of the rubric. This could have resulted in confusion on the meaning of each item on the rubric as well as a lack of understanding on faculty expectations of each level of competence. It is likely that this oversight may have resulted in the significant mismatch between the SP/TA assessments versus the students’ self-assessment of their skills and abilities. Future iterations will include student training on the rubric as well as examples of various levels of competency. 

When the students utilized MI techniques during their consultations, the data show improved self-confidence in skills. The data support the idea that targeted training can improve student confidence, overall counseling skills, and can be a useful exercise in self-awareness and self-evaluation. Providing students with the opportunity to assess their strengths and weaknesses are imperative to the development of any skill. 

Although this study demonstrated a positive impact on SED with the incorporation of MI training, there were some limitations within this study worth mentioning. Time and resources were the most significant limitations given the class size of over 200 students. Students received smoking cessation training as a 4 h online self-study module, MI training through a 3 h online self-study module, and then participated in a total of two simulated encounters. Due to this time constraint, students were only able to practice and develop the critical aspects of each without going into extensive detail and training. Self-study and self-paced modules have some advantages and disadvantages, most notably lack of student engagement. Frequent self-checks are built into the training to increase participation. The skills required to execute smoking cessation counseling and MI successfully requires multiple interactions to become proficient. In the future, we hope to incorporate the skills learned from these training longitudinally into the curriculum to gain the experience needed. 

Funding is also a limitation and required the use of SPs. Funding for this exercise was provided as an internal grant by our department, but to ensure long-term sustainability, we will need to find additional sources of funding to increase the number of simulations. Additionally, there appeared to be significant variation in grading between the SPs and TAs despite similar training. However, as mentioned above, this could be considered an asset when trying to develop students own social–emotional skills. Feedback and collaboration from experts in the area of social–emotional skills should be sought to enhance the development of this skill. Despite these limitations, this study provides insight into the importance of how SPs and MI can help students develop SEC. 

Further studies should explore the questions of what are the most effective ways to develop a students’ social and emotional skills and should MI training be a core requirement of a pharmacy school curriculum to improve counseling and communication skills. Medical schools are already trying to answer the latter question. The University of Virginia School of Medicine curriculum currently includes required MI training in the first year of the program [8]. The need to incorporate MI training in a pharmacy school curriculum is essential to train future health care providers to excel in patient-centered encounters, focus on individual patient needs, and elicit health behavior change. This study supports the use of patient simulations to develop students’ communication skills, MI skills, and overall SED. The results of this study showed that even in a short two-week period, students’ competency and confidence in their SEC and MI counseling skills improved significantly. Ultimately, longitudinal studies using actual patients to assess clinical outcomes would be best to determine if students trained in MI were effective in changing patients’ behavior. 

## 5. Conclusions

This study aimed to look at MI as an educational intervention to help develop a students’ social and emotional development. MI training showed significant improvement in students’ competency in social–emotional, communication, and MI skills. Additionally, increased confidence in students’ self-assessed skills and abilities were also noted. 

## Figures and Tables

**Table 1:** Post-survey student responses.

Post Survey Question	*N*	Mean	SD	SEM
On a scale from 1 to 5 (1–5) indicate your level of agreement with each of the following statements:				
1. The SP did a good job of creating a realistic scenario.	186	4.57	4.57	0.34
2. The SP did a good job creating a believable character.	186	4.58	4.58	0.34
3. he feedback the SP provided was constructive.	186	4.58	4.58	0.34
4. The simulation improved my abilities in counseling a patient on smoking cessation.	186	4.60	4.60	0.34
5. The feedback from the SP was fair.	186	4.54	4.54	0.33
6. The feedback I received from the SP today has made me aware of how a patient could perceive me in a healthcare setting.	186	4.54	4.54	0.33
7. The SP was effective in helping me understand how to be more socially and emotionally competent.	186	4.55	4.55	0.33
8. The SP today was effective in giving me feedback on my professional communication skills.	186	4.52	4.52	0.33
9. The SP today was effective in giving me feedback on my social and emotional development.	186	4.46	4.46	0.33
10. This simulation, focusing on professional communication with a patient, should be offered each year.	186	4.50	4.50	0.33
Based on a scale from 1 to 5 (1–5), how satisfied were you with the following?				
11. The self-study module on motivational interviewing	186	3.91	3.91	0.29
12. Simulated smoking cessation patient counseling sessions.	186	4.26	4.26	0.31
13. SP feedback on your performance.	186	4.35	4.35	0.32
On a scale from 1 to 5 (1–5) indicate your level of agreement with each of the following statements:				
14. I am more aware of the social–emotional component of patient counseling.	186	4.41	4.41	0.32
15. The motivational interviewing training will positively impact my future approach to patient counseling.	186	4.33	4.33	0.32
16. This simulation improved my abilities in providing effective patient counseling.	186	4.41	4.41	0.32
